# Laser-Induced Deposition of Carbon Nanotubes in Fiber Optic Tips of MMI Devices

**DOI:** 10.3390/s19204512

**Published:** 2019-10-17

**Authors:** Natanael Cuando-Espitia, Juan Bernal-Martínez, Miguel Torres-Cisneros, Daniel May-Arrioja

**Affiliations:** 1 CONACyT, Applied Physics Group, DICIS, University of Guanajuato, Salamanca, Guanajuato 368850, Mexico; 2 Unidad de Investigación Biomédica y Nanotecnología, Calle Cañada Honda 129, Ojocaliente 1 Aguascalientes, Ags. C.P. 20190, Mexico; 3 Applied Physics Group, DICIS, University of Guanajuato, Salamanca, Guanajuato 368850, Mexico; torres.cisneros@ugto.mx; 4 Centro de Investigaciones en Óptica, Prol. Constitución 607, Fracc. Reserva Loma Bonita, Aguascalientes 20200, Mexico; darrioja@cio.mx

**Keywords:** optical fibers, carbon nanotubes, multimode interference, laser-induced deposition

## Abstract

The integration of carbon nanotubes (CNTs) into optical fibers allows the application of their unique properties in robust and versatile devices. Here, we present a laser-induced technique to obtain the deposition of CNTs onto the fiber optics tips of multimode interference (MMI) devices. An MMI device is constructed by splicing a section of no-core fiber (NCF) to a single-mode fiber (SMF). The tip of the MMI device is immersed into a liquid solution of CNTs and laser light is launched into the MMI device. CNTs solutions using water and methanol as solvents were tested. In addition, the use of a polymer dispersant polyvinylpyrrolidone (PVP) in the CNTs solutions was also studied. We found that the laser-induced deposition of CNTs performed in water-based solutions generates non-uniform deposits. On the other hand, the laser-induced deposition performed with methanol solutions generates uniform deposits over the fiber tip when no PVP is used and deposition at the center of the fiber when PVP is present in the CNTs solution. The results show the crucial role of the solvent on the spatial features of the laser-induced deposition process. Finally, we register and study the reflection spectra of the as-fabricated CNTs deposited MMI devices.

## 1. Introduction

Engineered devices based on carbon nanotubes (CNTs) represent one of the most promising and active areas of nanotechnology development. Since its discovery, CNTs have attracted the attention of the scientific community due to the extraordinary properties exhibited by these materials. In particular, their electrical, chemical and optical properties have been exploited to develop electrochemical devices [1,2,3,4,5], photonic and laser applications [6,7,8,9,10] and optical biosensors [11,12,13] to name a few. Moreover, CNTs have been used as sensing material in pressure [14], flow [15,16] strain [17] and protein sensors [18]. The deposition of CNTs on the surface of optical fiber tips allows for the integration of the unique properties of CNTs into robust and versatile photonic devices. Chemical vapor deposition (CVD) [19], drop-casting [20,21], and Langmuir-Blodgett deposition [22,23] are some of the conventional techniques reported to incorporate CNTs into fiber optic tips. More recently, light-induced deposition has been demonstrated to generate saturable absorbers for ultra-short pulsed lasers [24,25]. Light-induced deposition is an inexpensive and reliable technique which has been also used to develop micro heaters and microbubble generators [26,27]. In order to obtain a CNTs deposit onto the tip of a fiber, a near-infrared (NIR) optical source is used to launch an optical power of 30–100 mW to a fiber tip immersed into a CNTs solution. According to previous reports, a thermal gradient attracts the CNTs to the tip of the fiber generating a deposit of the nanostructures at the core of the fiber [27].

In contrast with conventional techniques, light-induced deposition can be controlled by means of few experimental parameters such as wavelength, power and irradiation time. Moreover, as this technique relies on thermal gradients at the fiber-liquid interface, it is reasonable to think that the specific characteristics of the liquid solution may affect the performance of the deposition technique. However, very limited information can be found on the effects of different solutions over the light-induced deposition of CNTs. Moreover, the laser-induced deposition of CNTs has been demonstrated mainly in single-mode fibers as the specific features of nanotubes are expected to occur at localized spatial regions [24,25]. Laser-induced deposition on different fibers and fiber structures are yet to be demonstrated which may lead to attractive features in the areas of sensing and opto-fluidics. For example, multimode interference (MMI) fiber sensors are low cost, easy to fabricate and highly stable devices with adequate capabilities to be used as pressure [28,29], temperature [30,31], refractive index [32,33] and liquid level sensors [34,35]. Although the incorporation of carbon nanostructures into an MMI fiber device has been addressed previously [36], the method used to achieve carbon nanostructures deposition onto the optical fiber was drop-casting; leaving laser-induced deposition method yet to be investigated in the framework of MMI structures.

In this study, we explore the laser-induced deposition method to obtain CNTs deposits onto the fiber tips of MMI devices. The aim of this work is to demonstrate laser-induced CNTs deposits into MMI structures and to study the influence of different CNTs solutions on the as-fabricated deposits. These two specific aims will help in the design and development of future MMI sensors in which the spectral and spatial features of the CNTs deposits will be of relevant importance. In particular, we investigate the feasibility of laser-inducing CNTs deposits onto the tip of an MMI structure comprised of a single-mode fiber (SMF) spliced to a section of no-core fiber (NCF). Solutions of single-wall carbon nanotubes (SWCNTs) and multiwall carbon nanotubes (MWCNTs) in deionized water as well as in methanol were tested. In addition, solutions with a widely used surfactant (polyvinylpyrrolidone, PVP, [37,38,39,40]) were also studied. Moreover, PVP has been previously used as a surfactant in solutions of carbon nanostructures [41,42,43,44]. The laser-induced deposits were studied by means of optical microscopy and the reflected spectra of a broadband source which was registered by means of an optical spectrum analyzer (OSA).

## 2. Materials and Methods

The MMI fiber structure was built by splicing a segment of a commercially available silica-based no-core fiber (NCF) with refractive index of 1.444 (at a wavelength of 1550 nm) and diameter of 125 µm (Prime Optical Fiber Co. Ltd., NCF125) to a commercial single-mode fiber (SMF-28) using a Fujikura splicer (FSM S70). Then, the end of the no-core fiber was cleaved to obtain a spliced segment of the no-core fiber of 58.8 mm. The spectral response of the MMI structure depends on the geometrical and optical properties of the device as follows [45,46]:

(1) $$\lambda_{0}=p\left(\frac{nD^{2}}{L}\right),$$

Here, *λ*_0_ is the peak wavelength, *n* is the refractive index of the NCF, *D* is the diameter of the NCF, *L* is the length of the NCF segment and *p* is an integer. Briefly, in an MMI structure the initial single-mode launched from the SMF is coupled to all the available radial modes of the NCF and due to interference effects, the initial mode is refocused at a given length within the NCF. This phenomenon is known as self-image formation and occurs at a periodic length indexed by the integer parameter *p* in Equation (1). Moreover, Equation (1) relates the parameters of the NCF (refractive index *n* and diameter *D*) to the launched wavelength *λ*_0_ and the length *L* at which the *p-th* image is formed. Further details of MMI theory can be found elsewhere [32,47,48,49]. According to Equation (1) and for *p* = 4, the fabricated MMI device has a calculated peak wavelength of 1534.8 nm. Notice that the proposed MMI structure relies on the detection of reflected light at the end of the no-core fiber. After traveling a distance *L* over the no-core fiber, part of the light is reflected back and travels a distance *L* before coupling again to the SMF. In terms of spectral features, this is equivalent to a conventional transmission MMI structure with *p* = 8 and a length of 2*L*. In other words, the peak wavelength of an MMI device with a length *L* and for *p* = 4 is the same using Equation (1) with a length of 2*L* and *p* = 8. Moreover, it has been shown that the maximum coupling factor in MMI devices occurs periodically at every 4th image (i.e., *p* = 4, *p* = 8, *p* = 12) [32,50]. For a different wavelength, the self-image is formed at a different length which in turns decreases the corresponding coupling factor and ultimately provides MMI with filtering capabilities.

**Figure 1:**
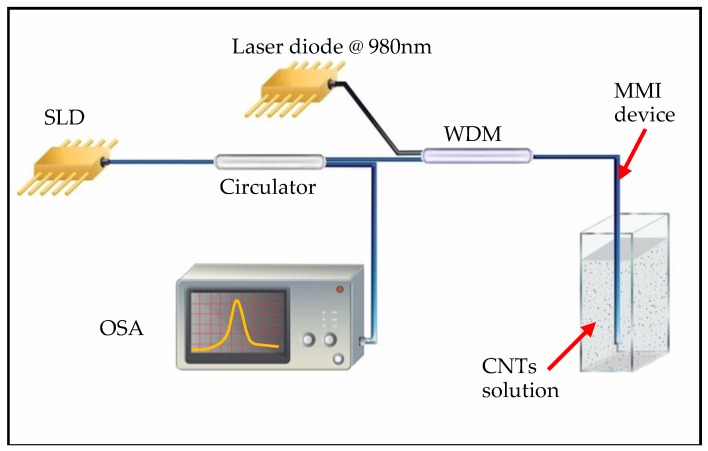
Schematic representation of the optical setup used in this study. SDL: superluminescent laser diode operating at 1550 nm with FWHM of 80 nm; WDM: wavelength division multiplexer; MMI device: multimode interference fiber device; CNTs solution: carbon nanotubes solution and OSA: optical spectrum analyzer. The depicted setup allows the fabrication of laser-induced deposition of CNTs and also the registration of the reflected spectrum of a broadband source.

In contrast with transmission-based MMI structures, reflection-based MMI structures are more suitable in sensing applications as the same fiber tip is used to send and collect light. After fabrication of the MMI structure, the fiber device was incorporated into an optical fiber setup which comprises a wavelength division multiplexer (WDM), an optical circulator, a laser diode and a superluminescent diode centered at 1550 nm. The experimental setup is shown schematically in Figure 1.

As Figure 1 shows, the WDM allows for launching light from two different sources to the MMI device. In particular, we have chosen a laser diode emitting at 980 nm as pump source for laser-inducing the CNTs deposits while we have used an SLD centered at 1550 nm to probe the reflection features of the as-fabricated CNTs deposits. The circulator depicted in Figure 1 allows for collecting the reflected light from the tip of the MMI device and analyzing the spectra with an OSA. A v-groove engraved steel plate was mounted on a translation stage to secure the position of the MMI device with a small circular magnet. Finally, a quartz cuvette is used to allocate the CNTs solutions and the MMI device is immersed into the CNTs solutions using the translation stage.

For the CNTs solutions, SWCNTs and MWCNTs were purchased from Cheap Tubes Incorporated (https://www.cheaptubes.com/). The length of the SWCNTs and the MWCNTs was in both cases reported by the manufacturer to be 200–500 nm. Outer diameter was chosen to be also similar; outer diameter of the SWCNTs used in this set of experiments was 1–2 nm according to the manufacturer. The outer diameter of the MWCNTs used in this study was reported by the manufacturer to be less than 8 nm. Previous reports have achieved light-induced CNTs deposits using polar solvents such as ethanol and dimethylformamide (DMF) [24,25,26,27]. Methanol was selected as polar solvent due to its simple chemical structure and its similar ability to form hydrogen bonds compared with water [51]. In order to explore the feasibility of generating CNTs deposits using a biocompatible solvent, solutions based on deionized water were also studied. The concentration of CNTs in the liquid solutions was kept fixed at 2.5 mg/mL for all the samples studied here. As one of the key factors when using CNTs solutions is the effective dispersion of the nanostructures over the liquid volume, we have added a widely used dispersant in the study. Since PVP is a polymer which has proved to be effective in dispersing CNTs solutions [41,42,43,44], a commercial PVP purchased from Sigma-Aldrich was used in all the experiments described here. A PVP concentration of 50 mg/mL was selected since this concentration has shown to be effective in dispersing CNTs in liquid solutions [43]. The general idea of adding a dispersant in laser-induced CNTs deposition experiments is to allow more homogeneous solutions and thus assisting in the realization of more homogeneous CNTs deposits onto the optical fiber end-faces. A total of 8 CNTs solutions were prepared as shown in Table 1.

For the experiments, the optical fiber is attached to the v-groove plate with a small magnet. Care was taken to place the magnet over a section of the SMF avoiding stress-induced features on the multimode fiber section of the device. Then, the MMI device is immersed in a set CNTs solution using a translation stage. Each CNTs solution was sonicated for 60 min before conducting the corresponding experiment. Once the MMI device is immersed, the laser diode is turned on to deliver a preset power to the tip of the MMI device for five minutes. After this period of time, the laser diode is turned off and the MMI device is taken out from the CNTs solution. Once air-dried, the tip of the MMI device is studied by means of optical microscopy. Finally, the SLD is used as a broadband source to probe the spectral features of the as-fabricated device.

## 3. Results

### 3.1. Laser-Induced Deposition of CNTs in Water Samples

**Figure 2:**
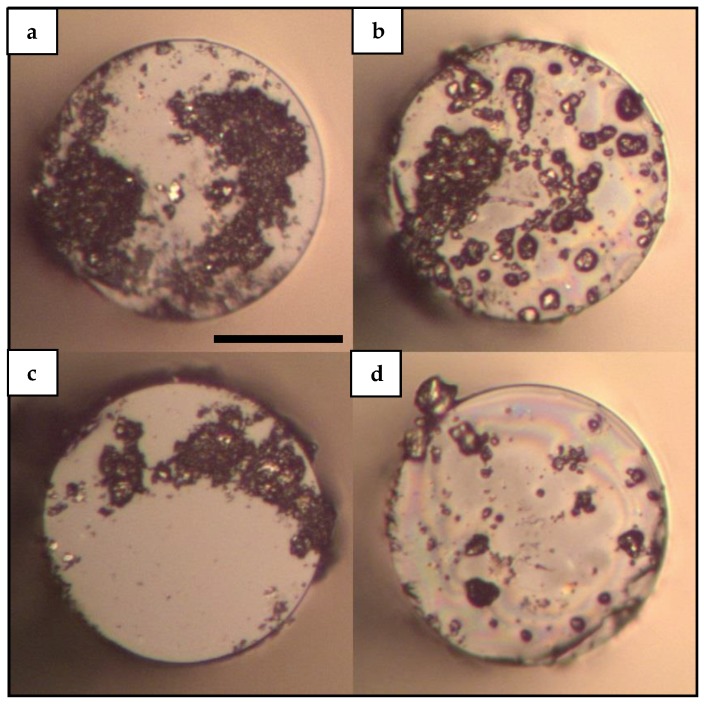
Laser-induced deposition of CNTs onto MMI fiber tip using water-based aqueous solutions: (**a**) Deposition performed with the W-SW sample; (**b**) Deposition performed with the W-SW-PVP sample; (**c**) Deposition performed with the W-MW sample; (**d**) Deposition performed with the W-MW-PVP sample. A 980 nm laser is used at 88.3 mW to obtain the laser-induced depositions in all cases shown in this figure. The images were taken using an optical microscope with a 20× microscope objective. The reference bar in panel (**a**) corresponds to 60 μm.

In general, water-based solutions of PVP/CNTs have been preferred as these solutions allow biocompatibility and stability for subsequent biomedical applications [42,43]. However, previous reports on the light-induced deposition of carbon nanostructures onto fiber tips have centered their attention on studying alcohol-based solutions of CNTs [24,25,26,27]. Figure 2 shows the tip of the MMI fiber device after the laser-induced deposition in water-based solutions of CNTs.

As Figure 2 shows, some accumulation of material is evident over the tip of the MMI device in all cases owing to some degree of CNTs deposition. However, the fiber tips exhibit non-uniform deposits when using water-based solutions of CNTs and the surface of the fiber tip is partially covered with CNTs material. According to MMI theory, the energy distribution of these fiber devices corresponds to radial-symmetric modes; which in turn concentrate the energy at the center of the fiber and in concentric ring-shaped structures [50]. The number of radial modes can be calculated as ~*V*/*π* for fibers with large *V* parameter [52]. In particular, for 980nm and the NCF used in this work the *V* parameter can be calculated as:

(2) $$V=2\pi \frac{a}{\lambda_{0}}\sqrt{n_{NCF}^{2}-n_{Water}^{2}},$$

In Equation (2), *a* is the NCF radius (62.5 μm), *λ*_0_ is the wavelength of the laser (980 nm), *n_NCF_* represents the refractive index of the NCF at 980 nm (1.45) and *n_Water_* is the refractive index of water (1.327). Equation (2) leads to a *V* parameter of 234.88 and thus a number of radial modes of ~75 which are enough to generate the characteristic ring structures of MMI effects. Nevertheless, the images of Figure 2 show no clear tendency of attached material around the center of the fiber or in ring-shaped structures. However, some differences can be extracted from the depositions using PVP in contrast to the depositions without PVP by analyzing the images shown in Figure 2. The corresponding deposits using water-based solutions with PVP (Figure 2b,d) show zones of attached material that exhibits smaller island-like features than their counterparts of deposits using water-based solutions without PVP (Figure 2a,c). These island-like structures on the deposits using PVP may suggest that the polymer is effectively reducing the agglomeration on the CNTs solutions during the deposition process.

### 3.2. Laser-Induced Deposition of CNTs in Methanol Samples

**Figure 3:**
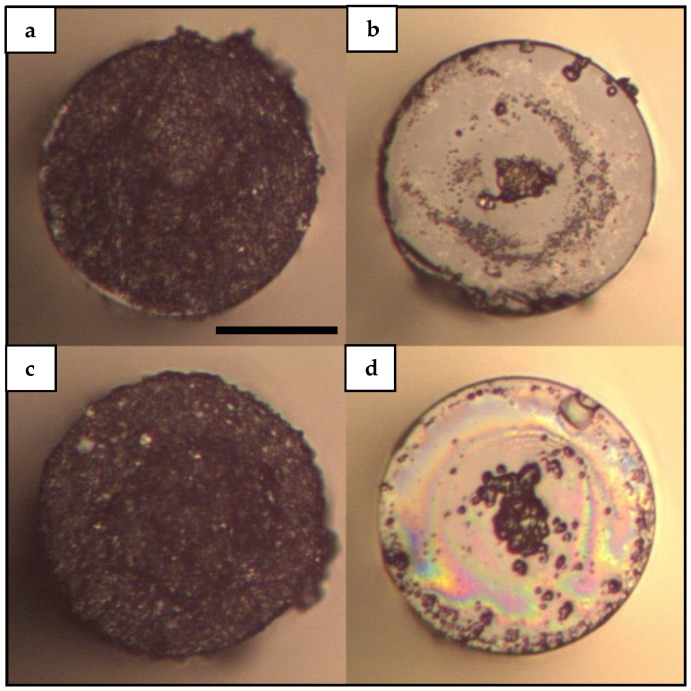
Laser-induced deposition of CNTs onto MMI fiber tip using methanol-based aqueous solutions: (**a**) Deposition performed with the M-SW sample; (**b**) Deposition performed with the M-SW-PVP sample; (**c**) Deposition performed with the M-MW sample; (**d**) Deposition performed with the M-MW-PVP sample. A 980 nm laser is used at 88.3 mW to obtain the laser-induced depositions in all cases shown in this figure. The images were taken using an optical microscope with a 20× microscope objective. The reference bar in panel (**a**) corresponds to 60 μm.

The images of the deposits on MMI fiber tips obtained with methanol-based solutions are presented in Figure 3. It is clear from Figure 3 that the behavior of the obtained deposits using methanol as solvent is essentially different than the deposits obtained with water-based solutions.

Figure 3a,c shows uniform and homogeneous CNTs deposits covering the surface area of the fiber tip. On the other hand, Figure 3b,d show images of deposits in which the material is found mainly around the center of the fiber. This result is a remarkable finding as to the difference between the left column (Figure 3a,c) and the right column (Figure 3b,d) is the use of PVP on the CNTs solutions. Figure 3b,d show similar island-like features as the ones observed in Figure 2b,d. In particular, Figure 3b exhibits scattered material on the surface of the fiber tip that resembles a ring-shaped structure. However, and comparing the upper and lower rows of Figure 3, it can be said that in general, deposits with methanol-based solutions of SWCNTs and MWCNTs present very similar behavior. This is important as this result shows that the proposed laser-induced deposition in MMI fiber devices allows the deposition of both SWCNTs and MWCNTs. The results shown in Figure 3 suggest that the action of the dispersant used in these experiments is not only related to promoting less agglomeration of carbon nanostructures, but also to a decrease in the interaction of carbon nanostructures with the surface of the fiber tip. In other words, as the presence of PVP in the solution reduces the interaction of the CNTs with the surface of the fiber, the deposits are formed at the center of the fiber where more energy is concentrated.

### 3.3. Deposition Features Dependence on Laser Power

**Figure 4:**
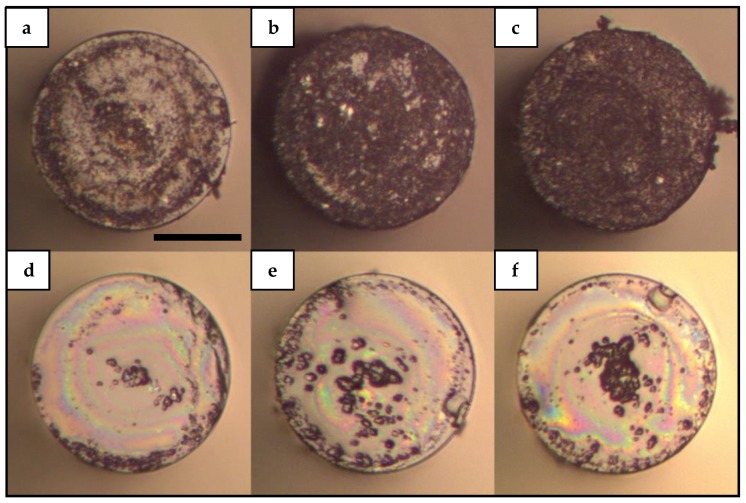
Laser-induced deposition of CNTs with different laser power: (**a**) M-SW sample at 40.0 mW; (**b**) M-SW sample at 88.3 mW; (**c**) M-SW sample at 139.1 mW; (**d**) M-SW-PVP sample at 40.0 mW; (**e**) M-SW-PVP sample at 88.3 mW; (**f**) M-SW-PVP sample at 139.1 mW. The images were taken using an optical microscope with a 20× microscope objective. The reference bar in panel (**a**) corresponds to 60 μm.

In order to study the effect of different laser powers on the proposed deposition technique, we performed laser-induced deposition experiments varying laser power. In particular, we ran laser-induced deposition as described previously with, 40.0, 88.3 and 139.1 mW. As expected, we found that as laser power increases the amount of material deposited on the fiber tip also increases. Figure 4 shows a series of images taken from depositions using M-SW and M-SW-PVP samples at different laser powers. Although Figure 4 shows images from depositions using M-SW and M-SW-PVP samples, we found that the increase of deposited material as laser power increases was present for all the samples studied here.

Figure 4a–c show homogeneous deposition on the fiber tip; similar to the depositions seen in Figure 3a,c. However, the images of Figure 4 show an increase of deposited material as laser power increases. Similarly, Figure 4d‒f show similar features to the images depicted in Figure 3b,d with an increase of deposited material at the center of the fiber as laser power increases. It is important to note that although the amount of deposited material increases with laser power in both cases, the deposition features with and without PVP are essentially different. For the upper row in Figure 4 (M-SW) the deposited material is found all over the surface of the optical fiber tip, while the lower row (M-SW-PVP) of Figure 4 shows accumulation of material around the center of the fiber. In other words, the distribution of the attached material over the surface of the fiber tip differentiates the deposits using aqueous solutions with and without PVP. It is clear from Figure 4 that while a homogeneous deposition over the tip of the fiber is allowed using CNTs solution without PVP, a central deposition of material is promoted with solutions including PVP. This essential difference in deposition features may indicate that the fundamental phenomena interplaying in the deposition processes are substantially altered with the presence of a dispersant such as PVP. It is important to note that although the NCF allows part of the energy to interact with the surroundings; Figure 2, Figure 3 and Figure 4 show that most of the CNTs material is attached to the fiber tips and very few CNTs material was observed to be attached along the outer surface of the fibers. This is related to the small amount of energy that interacts with the surroundings as the evanescent field compared to the energy leaving from the tip of the fiber. Notice also that the deposition of CNTs unto optical fibers has been associated with thermal gradients which are more difficult to induce only by means of evanescent fields propagating along the outer surface of the fiber. These interesting results can be useful in several areas such as fiber sensing and optofluidics where different devices can be engineered based on the specific distribution of the CNTs material onto the fiber tips. However, the exact mechanisms of the laser-induced deposition in optical fibers and the effect of dispersants on the processes governing the deposition of CNTs onto optical fiber tips are beyond this study.

### 3.4. Spectral Characterization of the CNTs Deposited MMI Fiber Probe

In order to evaluate the spectral changes of MMI devices after the laser-induced CNTs deposition process, we have recorded the reflection spectra of the proposed MMI fiber devices. As shown schematically in Figure 1, an SLD was used as a light source which provides a broadband spectrum of 80 nm of full width at half maximum (FWHM) centered on 1550 nm. An optical circulator and an OSA complete the fiber setup for spectral recording. Notice that the optical setup depicted in Figure 1 has been designed to take advantage of the fiber devices versatility to allow laser-induced deposition process as well as reflection sensing with a single optical setup.

Firstly, we registered the reflection spectrum in the air of a bare MMI device (*L* = 58.8 mm) with no CNTs deposition. The SLD was set at 100 mW for all the experiments. Then, the SLD was turned off, the MMI device was immersed in sample 1 solution and the 980 nm laser was then turned on to operate at 88.3 mW following the deposition process described previously. Notice that within this approach, the 980nm laser is used to induce the CNTs depositions while the SLD is used to probe the spectral features of the MMI device and both sources are not launched simultaneously. Upon drying, the corresponding spectrum is registered. Consequently, the MMI device is subjected to a multi-step cleaning/checking procedure. In the first step, the material attached to the outer surface of the fiber is removed by means of lens tissues and isopropyl alcohol. Then, the fiber is immersed in isopropyl alcohol and sonicated for 2 min. The isopropyl alcohol used in the previous step is then discarded and replaced with fresh isopropyl alcohol to sonicate again for 2 min. Once the fiber has been subjected to two sonication cycles, the tip of the fiber is evaluated by means of the optical microscope. If CNTs material or dust is found in the fiber, a two-cycle sonication is completed until the fiber is clean. Once no CNTs material or dust is found in the fiber, the reflection spectrum is measured again in order to corroborate the intactness of the MMI device. If the measured spectrum corresponds to the spectrum of the pristine MMI, the deposition using sample 2 is then performed and the test continues. However, if the spectrum has changed from the initial reference, the MMI is discarded and the fabrication process starts over. The test finished once the eight samples are effectively used to obtain CNTs depositions with the same MMI device. This procedure allows for a fair comparison of the spectra recorded.

**Figure 5:**
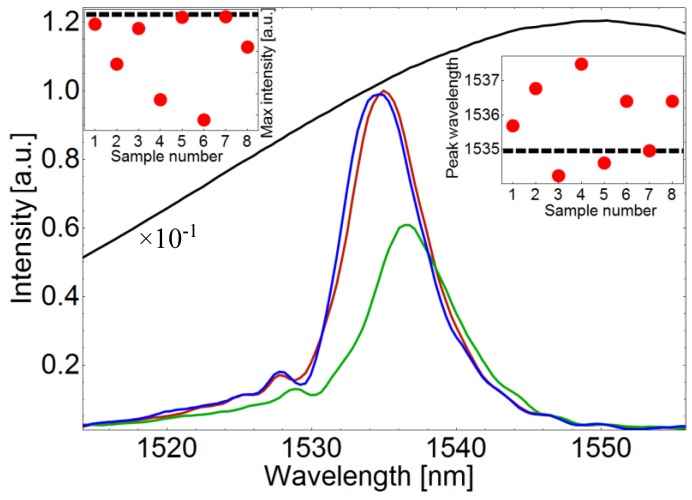
Spectral features of the as-fabricated MMI devices. The solid black line corresponds to the spectrum of the SLD source rescaled by a constant factor of 10^−1^ to emphasize spectral narrowing. The red solid line corresponds to the MMI device prior to CNTs deposition. Solid blue and green lines correspond to reflection spectra of CNTs deposited MMI device using M-SW and M-SW-PVP samples respectively. The left panel shows the maximum intensity as the function of the sample number used for the deposition experiments. The dashed line in the left panel corresponds to the maximum intensity of the spectrum of the bare MMI device. The right panel shows the peak wavelength as the function of the sample used in the laser-induced CNTs deposition experiments. The dashed line in the right panel corresponds to the peak wavelength of the bare MMI device.

Figure 5 summarizes the results of this set of experiments. The acquired reflection spectrum of the bare MMI device is shown in Figure 5 as a red solid curve. Figure 5 also shows a rescaled spectrum of the SLD source as a solid black curve. This spectral narrowing is one of the key features of MMI devices [28,29,30,31,34,35] with a peak wavelength that can be calculated according to Equation (1). The calculated peak wavelength for this device was 1534.8 nm which is very similar to the experimental peak wavelength of 1534.9 nm. As expected, a narrow spectrum (FWHM = 8 nm) was observed for all the depositions studied here. Moreover, a spectral shape with one dominant peak close to the wavelength of the bare MMI device was also found in all the reflection spectra recorded. In general, the intensity of the reflection spectra was found to be less for the case of the depositions using samples with PVP comparing to the depositions using samples without PVP. This is depicted in the left panel of Figure 5, where the maximum intensity (i.e., the intensity at the peak wavelength) is plotted as a function of the sample number. The dashed line in the left panel of Figure 5 represents the maximum intensity of the reflection spectrum of the bare MMI device.

In addition to the reflection spectra of SLD and bare MMI device, Figure 5 depicts two representative spectra of this set of experiments. Namely, the corresponding spectra of deposits obtained with M-SW and M-SW-PVP samples are depicted as solid blue and solid green lines respectively. As Figure 5 shows, the corresponding spectrum of the M-SW sample exhibits a small shift to shorter wavelengths without considerable loss in intensity. On the other hand, the corresponding spectrum of the M-SW-PVP sample shows an important shift to longer wavelengths with a considerable loss in intensity compared to the initial reflection spectrum. A possible explanation for this reduction in intensity can be a PVP film covering the surface of the fiber tip. In general, a film of material with a refractive index higher than the refractive index of air would decrease the contrast index and thus decreasing the reflectivity of the fiber probe. This scenario is further supported by observing the images from Figure 2, Figure 3 and Figure 4; in which rainbow-colored features are present in most of the depositions performed using solutions with PVP. However, this laser-induced PVP film has not been confirmed yet and more analysis is needed in order to clarify the nature of the rainbow-colored features and their relationship with a change in reflectivity in the tip of the fiber.

**Figure 6:**
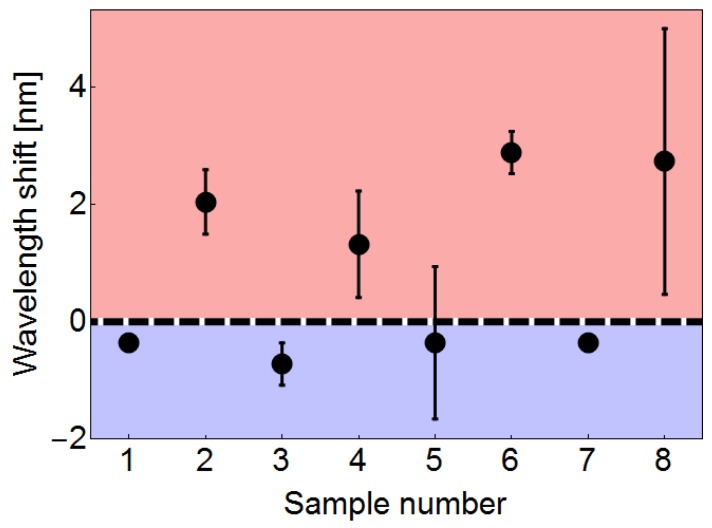
Wavelength shift as a function of sample number (see Table 1). Positive wavelength shifts represent a shift to longer wavelengths. Each symbol represents the mean value obtained from three MMI devices (see text). The bars represent the standard deviation.

The right panel of Figure 5 shows peak wavelength as a function of sample number for the as-fabricated deposits with an MMI device of 58.8 mm. The horizontal dashed line in the right panel of Figure 5 corresponds to the designed peak wavelength (i.e., the peak wavelength of the bare MMI device). In general, these results display a tendency in which the spectra from samples with PVP seem to shift to longer wavelengths while the corresponding spectra from samples without PVP seem to shift to shorter wavelengths. In contrast with a decrease in intensity in which the source can be modulated to compensate the losses, a shift in peak wavelength may drastically alter the resolution, accuracy and range of a fiber sensor based on this deposition technique. Therefore, more details of this particular behavior need to be addressed. In particular, the wavelength shift upon different designed peak wavelengths is important for future sensing applications. In order to explore this behavior, we have performed the proposed deposition technique on MMI fiber devices with designed peak wavelengths between 1529.6 and 1583.3 nm by allowing the no-core fiber length *L* to be 59–57 mm. In particular, MMI devices with *L* = 57, 58 and 59 mm were fabricated. Then, laser-induced deposition experiments using the samples shown in Table 1 were carried out as previously described for each of the MMI devices constructed. A fixed optical power of 88.3 mW was used for this set of experiments. Finally, the corresponding spectra were registered and the wavelength shift from the designed wavelength peak of the bare MMI device was obtained. The results of this set of experiments are shown in Figure 6.

In Figure 6, the wavelength shift has been defined as the difference between the experimental peak wavelength and the designed wavelength for the corresponding deposition experiment. Then, the horizontal dashed line in Figure 6 corresponds to the designed wavelength; a positive wavelength shift corresponds to a shift to longer wavelengths and a negative wavelength shift corresponds to a shift to shorter wavelengths. The results shown in Figure 6 indicate that the behavior of red/blue shifting with/without PVP is present in the deposition experiments regardless of the initially designed wavelength. These results provide important considerations for the proposed fiber devices in future sensing applications in which the peak wavelength of the reflection spectrum has to be carefully chosen.

## 4. Discussion

The systematic study of the laser-induced deposition of CNTs onto MMI fiber tips with different aqueous solutions has revealed interesting features that suggest the interplay of a variety of factors on the spatial and spectral features of the laser-induced CNTs deposits. One of the relevant findings has been the non-homogeneous deposits obtained with water-based solutions. Although the use of water solutions with PVP has been extensively reported as dispersant solutions for CNTs [37,42,43,44], we obtained deposits in which the attached material was found scattered over the fiber tip and partially covering the surface of the fiber tip as seen in Figure 2. These results indicate that the effect of the solvent on the CNTs is only one factor of the solvent in the complex process of laser-induced deposition. Moreover, these results indicate that the material properties of the solvent are critical for obtaining a homogenous deposition of the CNTs material onto the fiber tip. According to references [25,27], the deposition process of light-absorbing nanoparticles onto the fiber tip is attributed to thermal gradients and convective currents. As the light is absorbed by the CNTs, part of the energy is dissipated to the surroundings generating thermal gradients and convective currents over the solution volume. According to this description, a solvent with a higher density such as water would decrease current velocities as compared with a solvent with a lower density such as methanol. The irregular deposits found in water-based solutions may be related to irregular thermal gradients due to a higher density but also due to a less efficient thermal diffusion in the nanoparticle-water interface. The exact profile of the light-induced thermal gradient is not trivial as it depends on the material properties of the particles and solvent at nanometric scale. Nanofluidics has shown that at nanoscale, heat diffusion phenomena may greatly differ from their macroscopic counterparts [53,54,55]. Moreover, it has been shown that the thermal conductivity of water at nanoscale is significantly dependent on temperature [56,57,58]. Although more experimental evidence is needed to unveil the rich processes behind the laser-induced deposition of nanostructures, the experimental results presented here show the pertinence of a detailed theoretical study in which the material properties of the solvent in laser-induced deposition experiments are adequately addressed.

On the other hand, depositions obtained with methanol solutions showed a clear difference when PVP was added to the CNTs solution. In a counterintuitive result, the homogeneous deposits are obtained for solutions without PVP. This result can be explained taking into account the dispersion achieved by the intense sonication performed before the deposition and the fact that the deposition process takes a few minutes. However, the intriguing tendency of the CNTs material to attach to the center of the fiber for methanol-based solutions with PVP cannot be explained in terms of sonication or in terms of the initial dispersion conditions of the CNTs solution. As PVP has been used previously as the polymer matrix in optical-enhancing films on silica substrates [59,60,61,62], one possible scenario is a thin film of PVP covering the tip of the fiber preventing the direct interaction of the CNTs with the surface of the fiber. This film would also increase the optical energy needed to allow the deposition of CNTs material onto the fiber tip and thus enabling the deposition process mainly at the center of the fiber where the optical energy is concentrated. In addition, it has been shown that PVP promotes the dispersion of CNTs in aqueous solutions by adhering to the external surface of the CNTs structure [63,64,65,66]. This PVP wrapping may affect the amount of light absorbed by the CNTs and also may substantially alter the way the heat is diffused into the solvent volume. Again, the exact photothermal processes are beyond the aim of this experimental study as the objective of this work is to serve as the starting point in the design of MMI fiber sensors based on the laser-induced deposition of CNTs.

Finally, we have found that regardless of the solvent or CNTs structure used, the reflection spectra from depositions using solutions with PVP present a wavelength shift to longer wavelengths. This may reinforce the idea of a thin PVP film on the fiber tip as MMI fiber devices have been used to interrogate the refractive index of a liquid by analyzing the spectral response of the fiber device [32,33,34,35,67]. As the peak wavelength of any fiber sensor is one of the key sensing parameters, the shift induced by the deposition process must be taken into account when designing a fiber sensor based on the proposed deposition technique. In general, the results presented here demonstrate the feasibility of a fiber sensor based on laser-induced CNTs deposition. The use of a biocompatible optical fiber in combination with a reflection geometry represents attractive features for biomedical applications. Moreover, the use of functionalized CNTs within this approach will complete the reference frame to design and construct high-sensitivity fiber sensors with custom spatial and spectral deposition features.

## 5. Conclusions

We have demonstrated the laser-induced deposition of CNTs onto an MMI fiber device. Compared to conventional deposition methods, the laser-induced method represents a cost-effective, straightforward yet versatile and robust technique to obtain CNTs deposition onto optical fiber tips. The characteristics of the solvent in the CNTs solutions have shown to be crucial on the spatial and spectral features of the depositions studied here. The results presented here may lead to micro-patterned deposition of CNTs based on modal laser control. Depending on the spatial laser profile and the solvent used in this technique, a heterogeneous multistep CNTs deposition can be envisioned allowing deposition of CNTs with different features over the tip of the fiber. The structures presented here represent a robust platform to sense the change in the optical properties of the deposited CNTs. For example, a variation on the optical absorbance of the deposited CNTs would be tracked as an intensity variation on the reflected light at the fiber tip. As the peak intensity of the spectral features in these devices would be related to the change of CNTs optical properties, functionalized and conjugated CNTs [68,69] are ideal candidates to be incorporated into the presented methodology to develop high-sensitivity fiber sensors. In particular, pH-sensitive CNTs have been reported in which the absorbance of CNTs solutions is modulated through the pH of the solvent in which the CNTs are immersed [70]. Moreover, and due to the intrinsic spectral shift sensitivity of MMI devices on the surrounding refractive index, the structures presented here would help to develop multivariable/multiplexed sensors by relating a peak wavelength shift with the refractive index of the liquid in contact with the outer surface of the device. Finally, future work on the presented approach utilizing functionalized CNTs to detect specific targets will be particularly important for biomedical applications.

## Figures and Tables

**Table 1:** Aqueous samples studied in this report. For all cases, the CNTs concentration was 2.5 mg/mL. The samples with PVP were prepared at a concentration of 50 mg/mL.

Sample Number	Description	Abbreviation
1	Deionized water + SWCNT	W-SW
2	Deionized water + SWCNT +PVP	W-SW-PVP
3	Deionized water + MWCNT	W-MW
4	Deionized water + MWCNT + PVP	W-MW-PVP
5	Methanol + SWCNT	M-SW
6	Methanol + SWCNT + PVP	M-SW-PVP
7	Methanol + MWCNT	M-MW
8	Methanol + MWCNT + PVP	M-SW-PVP
